# Supplementary material for: Causal interplay between lactose intolerance and gut microbiota: a combined bidirectional Mendelian randomization and in vivo validation study
Source: Front Nutr. 2026 Jun 1;13:1803337. doi: 10.3389/fnut.2026.1803337 (PMC13265576; doi:10.3389/fnut.2026.1803337)
Supplement: Supplementary file 1 [file Data_Sheet_1.zip › supplementary materials/Forward/leave one out cross validation/ebi-a-GCST90027827.finngen_R12_E4_LACTONAS.pdf]

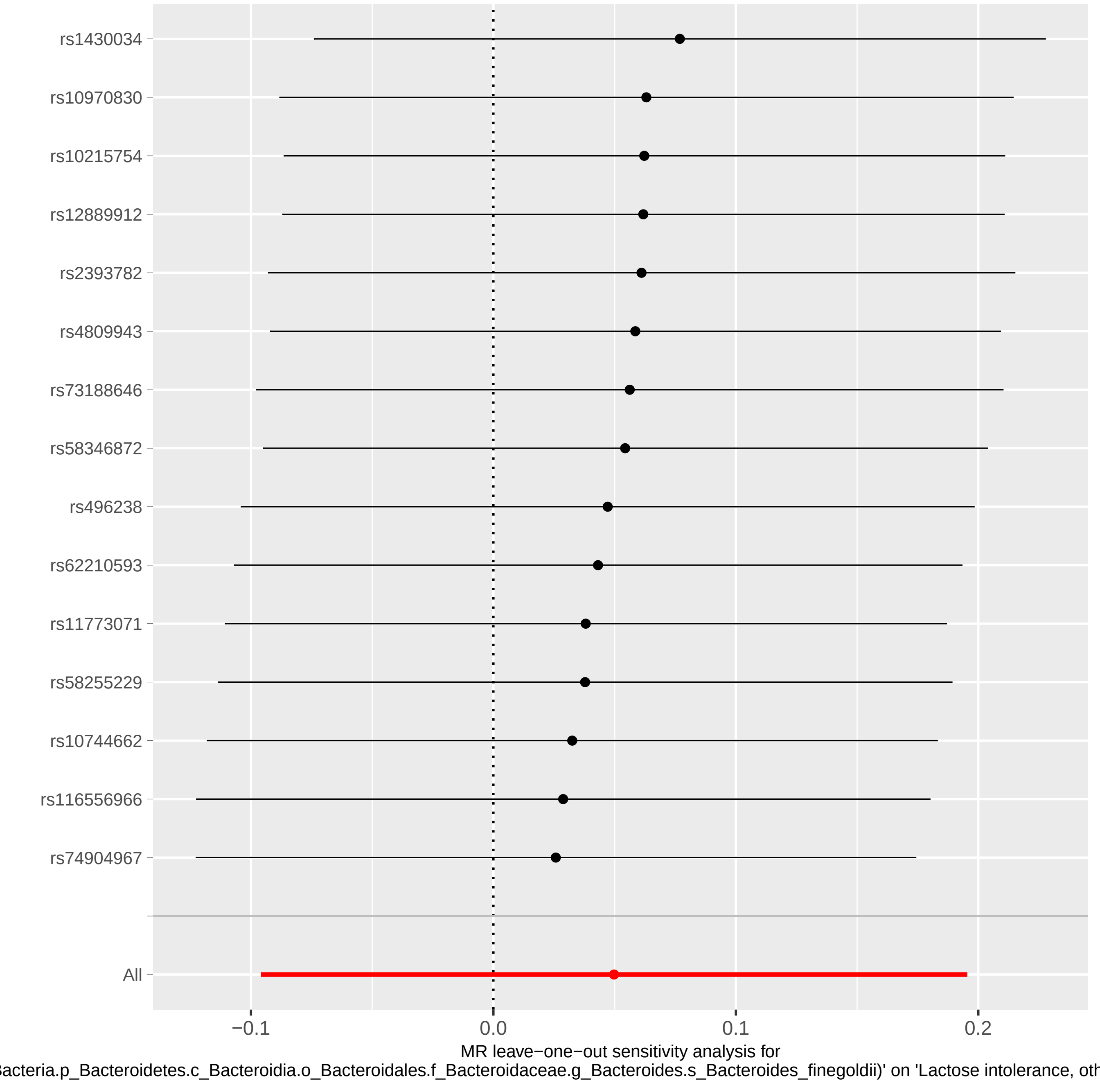
rs1430034
rs10970830
rs10215754
rs12889912
rs2393782
rs4809943
rs73188646
rs58346872
rs496238
rs62210593
rs11773071
rs58255229
rs10744662
rs116556966
rs74904967
All
−0.1
0.0
0.1
0.2
MR leave−one−out sensitivity analysis for
Bacteria.p_Bacteroidetes.c_Bacteroidia.o_Bacteroidales.f_Bacteroidaceae.g_Bacteroides.s_Bacteroides_finegoldii)' on 'Lactose intolerance, oth
